# Respiratory Tract and Eye Symptoms in Wildland Firefighters in Two Canadian Provinces: Impact of Discretionary Use of an N95 Mask during Successive Rotations

**DOI:** 10.3390/ijerph192013658

**Published:** 2022-10-21

**Authors:** Nicola Cherry, Natasha Broznitsky, Mike Fedun, Tanis Zadunayski

**Affiliations:** 1 Division of Preventive Medicine, University of Alberta, Edmonton, AL T6G 2T4, Canada; 2 BC Wildfire Service, Ministry of Forests, Victoria, BC V8T 5J9, Canada; 3 Alberta Wildfire Service, Agriculture and Forestry Alberta, Edmonton, AL T5S 1L3, Canada

**Keywords:** wildland firefighters, respiratory symptoms, respiratory protection, N95 masks, intervention

## Abstract

We examined whether discretionary use of an N95 mask reduced symptom reporting in wildland firefighters. The study collected data from two Canadian provinces during the 2021 fire season, with each firefighter followed for up to 4 rotations. Participants completed questionnaires on symptoms at the start and end of each rotation, when they reported also on mask use (if any) and completed a task checklist. Eighty firefighters contributed data. Nineteen firefighters were successfully fit-tested for N95 masks to wear whenever they felt conditions justified. Start-of-rotation symptoms reflected total hours firefighting in 2021. Symptoms of eye, nose and throat irritation and cough were more bothersome at the end of rotation. Cough, throat and nose (but not eye) symptoms were reported as significantly less bothersome at the end of rotation by those allocated masks, having allowed for crew type and start-of-rotation symptoms. Among those allocated a mask, use was most frequent during initial attack and least during driving and patrol. Reasons for not wearing included high work difficulty and low comfort. It is concluded that symptoms in wildland firefighters increased with hours of exposure. While provision of an N95 mask reduced symptoms, work is needed to overcome barriers to respiratory protection

## 1. Introduction

Studies of wildland firefighters suggest that lung function deteriorates and respiratory symptoms increase during the course of the fire season. Adenona et al. [1], in a review of 16 publications on short-term health effects in wildland firefighters, concluded that occupational wildland smoke exposure may have a cumulative negative effect on lung function. As evidence, the authors quoted six studies that measured lung function at the start and end of the fire season and documented decline in FVC, FEV1 and/or FEV1/FVC: it was unclear whether such decline persisted during the months away from firefighting between fire seasons. Rothman et al. [2] but not Betchley et al. [3] found a cross- season increase in respiratory symptoms that were most in evidence immediately post fire [4]. In North America, wildland firefighters seldom wear respiratory protection, although bandanas may be worn in an attempt to reduce smoke irritation. There is a perception that wearing respiratory protection would increase heat stress and decrease work capacity [5]. In a pilot study [6] wildland firefighters were randomly allocated to wear no respiratory protection, a half facepiece mask with P100 organic vapor cartridge, or a disposable N95 mask during a single shift. In this small sample, N95 masks (*N* = 9) were worn for a greater proportion of the shift than the half facepiece mask (*N* = 10) and were no less effective in reducing polycyclic aromatic hydrocarbon (PAH) absorption and cough. The current study was set up to assess the effect of the discretionary use of an N95 mask on respiratory tract symptoms and on PAH absorption, as indicated by urinary 1-hydroxypyrene (1-HP). As PAHs are absorbed through the skin, as well as the respiratory tract, the effect of enhanced skin hygiene on urinary 1-HP was also considered. The initial protocol was to collect baseline data at the start of the fire season, before exposure and to assess the effect of the skin hygiene and masks throughout the season. The modified protocol described here was adopted part way through the 2021 fire season when the lifting of COVID-19 restrictions made fieldwork possible. The effect of the two interventions (enhanced skin hygiene and allocation of masks) on urinary 1-HP has been reported elsewhere [7]. The present report considers whether wildland firefighters were increasingly bothered by irritant symptoms (eyes, nose, throat, cough) over single and successive rotations during the fire season, and whether the discretionary use of an N95 mask reduced such symptoms.

## 2. Materials and Methods

### 2.1. Recruitment and Assignment to Interventions

Fire crews in Alberta and British Columbia (BC) were identified who were willing to take part in a repeated measures study over successive rotations and to be randomly allocated to normal practice, enhanced skin hygiene or enhanced skin hygiene with provision of N95 masks. These masks, which gave protection against particles but not gases, were to be used, at the discretion of the firefighter, as respiratory protection under high smoke conditions. The plan was to follow each crew for up to 4 rotations. In Alberta 6 crews were initially identified, five helitack crews (small crews flown in by helicopter to attack new fires) and one ‘unit crew’, a larger group of firefighters moving between fires to contain and extinguish them. A further Alberta unit crew was included later in the fire season, following a direct request from the crew members to join the study. In BC the plan was to follow one large unit crew over successive rotations, but the unit crew initially identified was involved in a disastrous fire immediately after recruitment and withdrawn from firefighting. A second unit crew was selected and followed for successive rotations, with some members of the original crew also contributing information later in the season. In Alberta, crews were allocated randomly, using a coin toss, within forest area to interventions (normal practice/skin hygiene/masks) by crew, with all members within a crew following the same regime. In BC, crew members were allocated, using random numbers, to an intervention with individuals with different regimes working side by side. Rotations in BC lasted from 6 to 14 days. In Alberta they were from 10 to 21 days but could be longer if the fire conditions demanded. Although operating from a home base, a firefighter could be moved with their crew (or occasionally as an individual) to fight fires elsewhere in the province, or be ‘exported’ to another province. When a crew was exported, it was generally not possible to carry out measurements on a fire-day. The 2021 fire season had few major incidents in Alberta but was exceptionally active in BC.

### 2.2. Interventions

The study was designed with two interventions, enhanced skin hygiene and provision of an N95 mask. Only the mask intervention is considered in this report. Crews and, in BC, individuals within crews allocated to wearing a mask were qualitatively fit-tested with a range of N95 masks at the time of consenting to the study and allocated the N95 mask that fitted best. The firefighter was provided with a supply of N95 of the appropriate size and brand to allow them to put on a clean mask whenever they encountered smoky conditions. They were to make this judgement for themselves, rather than waiting for an instruction. In BC allocation to intervention group was random. In Alberta allocation of helitack crews was also random but members of the initial unit crew were unwilling to be allocated to mask wearing and the other unit crew, joining late, continued without masks.

### 2.3. Fireline Records

With agreement from each participant, fire services from Alberta and BC made available fire records for the whole fire season, allowing us to document the amount of fire activity before recruitment to the study and during the period of study. In Alberta this information referred to hours worked in fire control activities during each rotation. In BC the unit reported was days. In BC fire records were maintained by the participant themselves and were not always available. In order to permit comparison across provinces the days reported in BC fire records were transformed to hours. Time firefighting was estimated for the whole fire season and to the start of each rotation.

### 2.4. Data Collection

Crew members completed a questionnaire at the start and end of each rotation. End of rotation questionnaires for crews on export were collected when the crew arrived back in Alberta, up to 3 days after they had been engaged in firefighting. Both at the start and end of rotation, participants were asked to record, using a visual analogue scale, how much they had been bothered by four symptoms (irritation to the eyes, nose, throat and cough) in the 24 h before completing the questionnaire. The questionnaire also asked about activities other than firefighting that might entail irritant exposures (smoking of cigarettes, being with smokers, gathering round a barbeque or fire pit) and the number of days since they had last worked on a fire. The end of rotation questionnaire included questions about the firefighting tasks carried out during that rotation. It also asked those allocated a mask the proportion of days on which they had worn the mask. In addition they were asked to estimate the number of days on which they did not wear it at all. Further, it asked how often they had worn the mask during each of 10 listed tasks that they had carried out during that rotation. Those who had ever worn the mask at any time during that rotation were asked to rate their experience with the mask on visual analogue scales reflecting comfort, fit and the extent to which it made breathing difficult, work as a firefighter more difficult and how far they felt protected. The questionnaires are included as Supplementary Materials SA.

### 2.5. Computation of Time Wearing Masks

Those allocated to wearing a mask reported wearing it never or almost never, less than half the days during the rotation or at least half the days. For the 10 tasks listed, all firefighters assigned to wearing a mask who reporting doing that task during the rotation were assigned a mask use score. This was zero for those who did not wear the assigned mask while carrying out that task and ranked to 100 for a firefighter who reported always wearing the mask doing that task.

### 2.6. Exposure to Irritant Particles and Vapors

Although air monitoring was carried out to assess the concentration of PAHs on particulates and in the vapor phase there was no measure of total particles or of airborne compounds or gases other than PAHs. Exposure to irritants may be inferred from the total hours of firefighting from fire-line records, and, during a rotation, to participation in an active, monitored fire (a ‘fire-day’).

### 2.7. Statistical Methods

As each firefighter appeared in the dataset for up to four rotations, a linear mixed effects model with robust standard errors was adopted, with responses clustered within firefighters. Characteristics of crew members were described and used to examine potential confounders of the symptom reporting. Eye and respiratory tract symptoms during the rotation were examined at the beginning and end of rotation for all firefighters and for those allocated to wear a mask, both by intention (assigned intervention) and reported practice. The relation of end of previous rotation symptoms to start of next rotation symptoms were examined to investigate the plausibility of cumulative effects over the season. Fireline records were used to estimate exposure since the start of the season to examine effects of cumulative exposure on start of rotation symptoms, using a model with random slope. Analysis was carried out in Stata 14.2. A probability *p* < 0.05 was used to indicate an effect unlikely to be due to chance.

## 3. Results

### 3.1. Participants and Fire Conditions

Table 1 gives the number of firefighters, rotations and monitored fire-days, broken down by the allocation to mask wearing. There were 10 firefighters in Alberta and 9 in BC assigned to wear an N95 mask (together with enhanced hygiene). These 19 firefighters contributed data from 49 rotations and were monitored on 35 fire-days. The eight firefighters from BC unit crew H who did not remain active with the crew (and are excluded from Table 1) all completed a start of rotation questionnaire and urine sample and are included where appropriate, increasing the number of participants to 80. Overall, 15/80 (19%) were female and this proportion was very similar between the provinces and type of crew (unit or helitack). The proportion, 66% (53/80) who had ever smoked at least one cigarette/day for at least a year was lower in BC unit crews (16/34; 47%) than in unit crews from Alberta (21/24; 88%). The crew members were largely in their mid-twenties (a median of 25 years) but ranged from 16–56 years of age. Those allocated to wear a mask did not differ from those not on mean age, gender or smoking status. The total firefighting recorded in fire-line records for the whole of the 2021 fire season in Alberta was very much greater for firefighters in unit crews (a median of 460 hours) than for the helitack crews (78 hours). No direct comparison could be made between unit crews from Alberta and BC as in BC firefighting activities were reported as days rather than hours. Exposure monitoring indicated a mean of 7.2 hours/day firefighting for unit crews in BC. This would suggest a median of 533 hours firefighting during the fire season for BC unit crews, 16% more hours than unit crews from Alberta. BC fires were recorded as larger and more complex, reflecting the difference in the ferocity of the fire season in the two provinces.

### 3.2. Symptoms at the Start and End of Rotations

The extent to which the participant was bothered by each of four symptoms (sore, itchy or running eyes; sore itchy or running nose; sore or rough feeling throat; coughing) was recorded on a visual analogue scale on which ‘not at all’ was coded as zero and ‘very bothered’ coded 100. Table 2 shows the mean start and end of rotation responses for the 4 symptoms within crew type. Both at the beginning and end of the rotation coughing was recorded as the most troublesome symptom overall but the rating of all four symptoms increased significantly from start to end. The table also shows that symptoms were importantly higher for unit crews, both at the start and end of rotation. Neither start nor end of rotation symptoms were related to age, a history of smoking or, in the previous 24 h, smoking cigarettes, spending time with people smoking tobacco, or spending time round a barbeque or firepit. Women in unit crews were more likely than men to be bothered by throat symptoms at the end of rotation, but this was not seen for other symptoms or at the start of rotation.

### 3.3. Evaluation of N95 Mask Allocation on Symptom Reporting

Overall, those who were allocated a mask reported being less bothered by symptoms at the end of rotation, having adjusted for start of rotation symptoms (Table 3). This was least evident for eye irritation and most for throat and cough symptoms. Examination of median scores, to allow for skew in the means, again showed marked differences with a median score of 0.8 for throat irritation and 1.7 for cough in those allocated masks compared to 14.4 for both throat irritation and cough in those not allocated. Table 4 shows the univariate relation of start of rotation symptoms, crew type and mask allocation to end of rotation symptoms and also multivariable models for these factors, for all firefighters and those taking part in a monitored fire-day. In the multivariable models (Table 4), eye symptoms at the end of rotation were not strongly related to starting eye symptoms, were higher in unit crew and were unrelated to mask allocation. Nose symptoms were reduced (with marginal significance) in those allocated a mask and whose activity was monitored as a fire-day. Both end-of-rotation throat and cough symptoms were reported as less bothersome in those allocated to discretionary use of an N95 mask.

Not all firefighters allocated a mask wore it. Among the 49 rotations carried out by those allocated a mask, an end-of-rotation questionnaire had been completed for 46. Of these, 19 said that they had worn the mask ‘never or almost never’. For some this may have been because there was little of no fire activity, but of 35 rotations with a monitored fire day (and so significant exposure) there were 11 where the firefighter reported little or no use of the mask. Those assigned a mask were asked to rate on a visual analogue scale from ‘Always’ to ‘Never’ the frequency with which they wore the mask while doing each of 10 listed tasks shown in Table 5. Not all firefighters did all the tasks during one rotation, but among those assigned a mask, initial attack had the highest mask use score and driving, patrol and hazard reduction the lowest. Table 3, which shows the effect of mask allocation on reported symptoms has been expanded to show (as Table 6) the relation of end of rotation symptoms to reported mask use among those allocated a mask. Those reporting seldom or never wearing the mask had fewer symptoms than those using it at least part of the time, but both were lower than those not provided with a mask.

Firefighters allocated a mask and who wore it at any time during that rotation were asked to rate their experience with the mask in that rotation, using a visual analogue scale (Table 7). This was completed for 34 of the 46 rotations with an end-of-rotation questionnaire. For three items (comfort, fit and protection) a high score (on a scale from 0–100) was favorable whereas on the other two (difficult breathing and firefighting work more difficult) a high score was unfavorable (see question D2 in the end of rotation questionnaire). Overall, on the scales where a high score was favorable, the perceived protection afforded by the mask received the highest mean score (66.3), followed by goodness of fit, with lowest scores for comfort (32.3). There was little difference between the scores given for making breathing difficult and making work difficult—both were, on average rated as showing agreement that the mask caused difficulties. Table 7 shows also the difference in scores by the extent to which the mask was worn (at least half the time, or less than half the time) and, as a quantitative measure, the number of days not wearing a mask that rotation (with a mean of 10 days). The likelihood of wearing a mask was lower in those who reported more difficulty breathing or working as a firefighter when wearing the mask. The number of days not wearing was significantly lower with increasing report of mask comfort and higher with increasing ratings of work difficulty. Both features contributed to the multivariate, multilevel model summarized at the bottom of Table 7. The 20 comments volunteered by the firefighters about their experience wearing the mask are given verbatim in Supplementary Materials SB. The comments were disparate, and included similar observation from the same firefighter on successive rotations. There was some feeling that masks were useful for mop-up but that they made verbal communication more difficult.

### 3.4. Accumulation of Symptoms over the Course of the Season

One initial aim of this study was to investigate whether there was any accumulation of symptoms over the fire season and whether this was reduced by wearing an N95 mask when conditions warranted. The late start to sample collection resulting from COVID-19 precautions meant that there were no data from firefighters before their first exposure in 2021. The earliest available were the start of rotation symptoms for the first rotation monitored in the study. We were able to calculate, from fire line records provided through the provinces, the number of hours/days engaged in active firefighting before the first sample for that rotation and to relate that to start of rotation symptoms. This cumulative time firefighting was related to symptoms reported on the first start of rotation questionnaire, and on all questionnaires completed at the start of a study rotation (Table 8). No evidence was found of modification of these effects by mask wearing once in the study.

**Figure 1:**
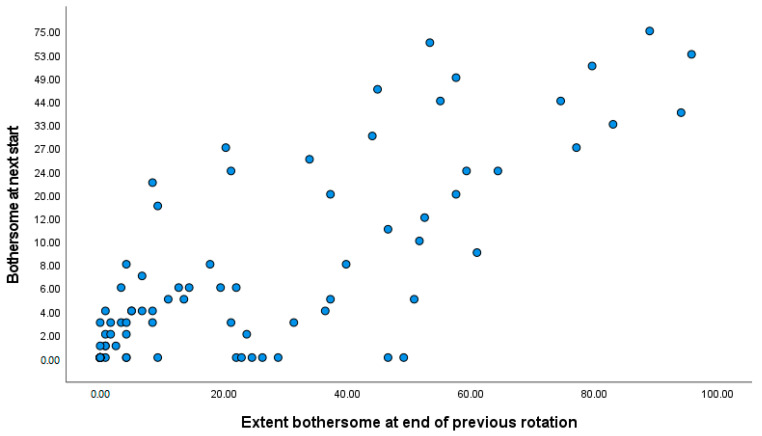
Plot of the extent to which cough bothersome at the start of the next rotation by extent bothersome at the end of previous rotation.

End of rotation symptoms (such as cough, Figure 1) strongly predicted symptoms at the start of the next rotation (Table 9), consistent with the relation between previous exposure and symptoms seen in Table 8. Days between questionnaires did not affect this observed relation.

## 4. Discussion

This report considers the effect of N95 masks, to be worn electively when conditions warranted. It has been reported elsewhere [7] that the allocation of a mask was associated with lower end of rotation urinary 1-HP, consistent with reduced PAH absorption. The analysis reported here suggests that mask allocation also reduced symptoms, particularly cough.

Importantly, it was left to the firefighter to determine when conditions were sufficiently bad to warrant wearing a mask and it is of interest that those reporting they never wore the allocated mask reported being less bothered than others by end-of-rotation symptoms. Use was emphasized to be discretionary and it may be that those who chose not to wear it truly saw no need. The conditions they encountered may have involved very little exposure to smoke: those in Alberta who did not have fire activity during a rotation reported low end of rotation symptom scores. Those reporting they had not worn an allocated mask may have chosen not to do so because they did not experience symptoms and so saw little point in using a mask: equally those with pre-existing respiratory conditions may have chosen to wear the mask more readily. Finally, we cannot dismiss the possibility that self-reported mask wearing and/or symptoms were inaccurate. Some may have used a mask but not reported it and others may have minimized symptoms. The study design is to assess the effect of allocation (‘intention to treat’) of a discretionary use mask and while reported practice is of interest, the key result is given in Table 3.

There are few data with which to compare these results. The increase in bothersome respiratory tract symptoms during a rotation and cumulatively during the fire season are consistent with the review by Adetona et al. [1] suggesting a cross-season decline in respiratory function, albeit without lung function testing in the current study. De Vos and colleagues [8] measured the effects on spirometry and symptoms of three types of mask during a two-hour prescribed burn exposure. They concluded that organic vapor filters were more protective than particulate filters against acute change in respiratory symptoms. We do not know of other data (except our earlier study [6]) assessing the use of masks in reducing respiratory symptoms in wildland firefighters. A recent scoping review did not identify additional studies [9]. The arguments against mask wearing center on the long-recognized increase in physiological demands on workers in physically demanding jobs, such as a wildland firefighter [10] and there has been concern that wearing a mask may give an unrealistic feeling of protection, resulting in a greater readiness to risk exposures. The need to mitigate exposures has been promoted [11] but centered largely on administrative controls for specific high exposure tasks [12,13] such as reducing time spent in mop-up and increasing speed of rotations through holding operations. Navarro [14] concluded that no respirator can provide protection from gases and particles for wildland firefighters performing physically demanding work. While an N95 mask is limited in the range of protection it can offer, its discretionary use may be more easily accepted by wildland firefighters than more cumbersome devices and, this study would suggest, may confer significant protection from some, but not necessarily all, smoke components.

A major limitation of the study was the absence of baseline data from the beginning of the season, before exposure. We have suggestive results, using fire line data, that respiratory symptoms became more bothersome with greater cumulative time firefighting. Baseline measures would have allowed stronger conclusions. Further, the crews studied had volunteered, and may not be representative of all firefighters. Strengths of the study include the ongoing collaboration of the firefighters over repeated rotations, with very few leaving before the end of the study, and an overall willingness to assist the study personnel in collecting samples and completion of questionnaires. Further, the availability of information from fire records for individual firefighters has only been touched on here. The wealth of data would be invaluable in assessing the relation of firefighting activities to chronic respiratory ill-health. Recent studies have reported differences in exposures between firefighting tasks [14,15]. Here, the analysis of discretionary mask use may give some indication of perceived smoke intensities, with mask use being greatest during initial attack and least while driving and patrolling.

The original protocol was designed to examine the effects of cumulative exposures during a fire season, and the impact of discretionary mask wearing during many consecutive rotations of firefighting. Such a study is still needed and would ideally have a full factorial design with random allocation of crews to interventions. Given the vagaries of ‘real life’ fieldwork [15] with, as here, COVID-19 restrictions together with calamitous fires in one province and few fires in the other, this would need major investment and organization. The conclusions from this study, with its several limitations, support the need for more effective intervention to reduce respiratory exposure, particularly for tasks carried out by unit crews. The N95 mask assigned here is a particulate respirator, considered by the US National Fire Protection Association Standard on Respirators [16] as suitable only for conditions in camp and away from fire combustion activities. The Standard lists requirements for air purifying masks (removing gases, aerosols and vapors) for operational wildland firefighters. As always, the best may be the enemy of the good, and given the current practice of no respiratory protection, the partial protection offered by a particulate respirator may be a worthwhile step while ways are found to overcome the significant barriers to full respiratory protection.

## 5. Conclusions

Respiratory tract symptoms in wildland firefighters increase with hours of exposure. Reports of symptoms may be reduced by the allocation on N95 masks, to be worn at the discretion of the firefighter.

## Figures and Tables

**Table 1:** Observations made in Alberta and BC during the 2021 fire season.

Crew Type	Rotations	Monitored Fire-Days	Crew on Export	Allocation of Mask		
				No	Yes	Total
Alberta						
Helitack A	3	1	0	4	0	4
Helitack B	2	1	1	4	0	4
Helitack C	3	1	0	0	6	6
Helitack D	3	1	0	4	0	4
Helitack E	2	2	0	0	4	4
Unit crew F	2	1	1	12	0	12
Unit crew G	1	0	1	12	0	12
Total Alberta	16	7	3	36	10	46
British Columbia						
Unit crew H	1	1	0	5	4	9 *
Unit crew I	4	4	0	12	5	17
Total BC	5	5	0	17	9	26 *
Total AB + BC						
All	21	12	3	53	19	72 *
Firefighters * rotations	164	101	28 **	115	49	164 *
Firefighters * fire days	-	101	-	66	35	101

* Unit crew H had 17 firefighters recruited for the study. Eight firefighters who left the service or did not take part in the fire-day are not included here. ** This include two rotations in which an individual BC firefighter was exported.

**Table 2:** Mean symptom scores at the start and end of rotation and crew type. How much have you been bothered by the symptom in the last 24 h (on a visual analogue scale 0–100)?

	Sore, Itchy or Running Eyes		Sore, Itchy or Running Nose		Sore or Rough Feeling Throat		Coughing		
Start of Rotation	Mean	SD	Mean	SD	Mean	SD	Mean	SD	N
Helitack crews Unit crews	1.57 12.05	2.19 18.59	3.40 10.97	7.60 18.12	2.57 12.31	3.91 17.68	3.60 14.41	6.26 18.40	58 111
All	8.45	15.90	8.37	15.73	8.96	15.21	10.70	16.17	169
**End of rotation**									
Helitack crews Unit crews	2.30 19.39	3.39 24.89	5.54 20.35	11.31 23.58	6.49 27.17	13.55 27.17	6.14 29.93	12.42 27.25	56 99
All	13.21	21.59	15.00	21.23	19.70	25.19	21.33	25.67	155
*p* * = Start: End	<0.001		0.001		<0.001		<0.001		

* probability from a linear mixed effects model allowing for repeat rotations.

**Table 3:** Allocation to mask wearing by end of rotation symptoms.

Assigned N95 Mask	Extent to Which Symptoms Were Bothersome							
	Eyes		Nose		Throat		Cough	
	Mean	SD	Mean	SD	Mean	SD	Mean	SD
No	15.21	23.96	17.12	22.03	22.77	25.60	24.90	26.38
Yes	7.72	13.59	8.53	16.79	10.58	19.66	11.83	19.46
Overall	12.99	21.65	14.58	20.93	19.16	24.57	21.03	25.19
N observations	152							
N firefighters	71							
*p* * =	0.236		0.038		0.012		0.010	

* Probability from a linear mixed effects model adjusted for symptom complaint at the start of rotation allowing for repeat rotations by the same firefighter and clustering within 9 crews.

**Table 4:** Relation of end of rotation symptoms to start of rotation symptoms, crew type, and allocated N95. Linear mixed effects model.

	Univariable			Multivariable					
				All			All with Fire Day		
	β	95% CI	*p*	β	95% CI	*p*	β	95% CI	*p*
Eyes									
Symptom at start	0.32	−0.34 to 0.98	0.338	0.30	−0.20 to 0.80	0.245	0.37	−0.19 to 0.92	0.198
Unit crew	15.09	8.63 to 21.55	<0.001	11.96	6.14 to 17.77	<0.001	11.19	4.91 to 17.46	<0.001
Allocated mask	−2.79	−11.28 to 5.71	0.520	−2.16	−7.45 to 3.14	0.424	−2.22	−8.63 to 4.19	0.497
Nose									
Symptom at start	0.57	0.29 to 0.84	<0.001	0.52	0.25 to 0.79	<0.001	0.59	0.35 to 0.83	<0.001
Unit crew	13.72	7.32 to 20.12	<0.001	9.39	3.63 to 15.16	0.001	7.43	−0.98 to 15.85	0.083
Allocated mask	−9.51	−17.04 to −1.97	0.013	−4.87	−10.88 to 1.14	0.112	−7.59	−15.87 to 0.68	0.072
Throat									
Symptom at start	0.61	0.32 to 0.90	<0.001	0.68	0.32 to 0.81	<0.001	0.60	0.35 to 0.84	<0.001
Unit crew	18.10	9.90 to 26.30	<0.001	11.82	4.34 to 19.30	0.002	14.00	5.36 to 22.64	0.001
Allocated mask	−11.27	−22.40 to −0.13	0.047	−8.54	−16.15 to −0.93	0.028	−8.80	−18.19 to 0.59	0.066
Cough									
Symptom at start	0.81	0.49 to 1.14	<0.001	0.69	0.41 to 0.96	<0.001	0.70	0.41 to 1.00	<0.001
Unit crew	23.61	13.52 to 33.71	<0.001	14.29	7.38 to 21.19	<0.001	13.24	3.92 to 22.57	0.005
Allocated mask	−15.41	−27.66 to −3.21	0.013	−7.22	−11.96 to −0.49	0.036	−9.23	−18.32 to −0.14	0.047
N									
Observations	155			152			97		
Firefighters	71			71			55		

**Table 5:** Mask use * by task in those assigned to wear a mask and carried out the task.

	All Assigned		
	Mean	SD	N
Initial attack	53.1	37.0	24
Sustained action	47.4	35.1	32
Prescribed fire	38.1	38.4	11
Hazard reduction	21.1	27.1	13
Hot spotting	45.5	35.6	34
Mop-Up	45.4	35.0	35
Burn out	36.0	31.5	13
Patrol—Recon	15.0	27.2	21
Gridding	36.1	40.1	23
Driving	9.6	21.0	24

* On a scale of 1–100, where 100 means worn all the time.

**Table 6:** Mask wearing by end of rotation symptoms.

Assigned N95 Mask?	Extent to Which Symptoms Were Bothersome								N Observations
	Eyes		Nose		Throat		Cough		
	Mean	SD	Mean	SD	Mean	SD	Mean	SD	
No	15.21	23.96	17.12	22.03	22.77	25.60	24.90	26.38	109
Yes: not worn	3.03	7.34	2.67	5.41	2.94	5.40	2.90	6.22	19
Yes: worn some or all of the time	11.42	15.89	12.93	20.35	17.36	24.46	18.39	22.66	27
All assigned	7.72	13.59	8.53	16.79	10.58	19.66	11.83	19.46	46

Observations with symptoms both at start and end of rotation.

**Table 7:** Visual analogue rating of 5 aspects of mask wearing by frequency of use and number of days mask not worn.

Mask Worn		Comfort	Fit	Protection	Breathing Difficult	Work Difficult	N
At least half the time	Mean	44.8	63.7	73.2	42.8	44.8	12
	SD	32.2	30.2	18.1	28.6	32.9	
Less than half the time	Mean	25.5	49.6	61.0	65.0	68.5	22
	SD	25.3	28.6	22.0	28.5	30.6	
Overall	Mean	32.3	54.6	66.3	57.2	60.4	34
	SD	29.0	29.5	21.2	30.1	33.0	
	*p* *	0.061	0.187	0.110	0.032	0.042	
Number of days mask not worn	β	–0.06	-0.01	–0.07	0.04	–0.05	
	95% CI	–0.09 to –0.03	–0.06 to 0.05	–0.14 to 0.00	–0.01 to 0.09	0.01 to 0.08	
	*p* *	<0.001	0.834	0.05	0.127	0.014	
Multi variate	β	–0.005	–	–		0.03	
	95% CI	–0.07 to –0.02				0.00 to 0.05	
	*p* **	<0.001				0.044	

* Probability from a linear mixed effects model allowing for repeat rotations. ** Probability from a multi-variate linear mixed effects model allowing for repeat rotations.

**Table 8:** Relation of estimated hours fire fighting since the start of the season to the extent to which symptoms were bothersome at the start of rotation.

	β	95% CI	*p* *	N **	
				Observations	Firefighters
Eyes	0.018	−0.00 to 0.04	0.076	138	66
Nose	0.031	0.02 to 0.04	<0.001	138	66
Throat	0.029	0.01 to 0.05	0.001	138	66
Cough	0.016	0.01 to 0.02	<0.001	138	66

* From a model with random slopes allowing for repeated rotations by the same firefighter and crew membership ** where fire line records were missing, no estimate could be made of total hours firefighting.

**Table 9:** Relation of start of rotation symptoms to symptoms at the end of the previous rotation (rotations 2–4 only).

	β	95% CI	*p* *	N	
Symptom				Observations	Firefighters
Eyes	0.178	0.08 to 0.27	<0.001	86	47
Nose	0.416	0.23 to 0.60	<0.001	86	47
Throat	0.337	0.10 to 0.57	0.003	86	47
Cough	0.448	0.33 to 0.56	<0.001	86	47

* *p* from a linear mixed effect model allowing for repeat rotations by the same fire fighter.

## Data Availability

Anonymized datasets may be made available in discussion with the corresponding author.

## Supplementary Materials

The following supporting information can be downloaded at: https://www.mdpi.com/article/10.3390/ijerph192013658/s1.
