# Supplementary material for: Respiratory Tract and Eye Symptoms in Wildland Firefighters in Two Canadian Provinces: Impact of Discretionary Use of an N95 Mask during Successive Rotations
Source: Int J Environ Res Public Health. 2022 Oct 21;19(20):13658. doi: 10.3390/ijerph192013658 (PMC9603074; doi:10.3390/ijerph192013658)
Supplement: Supplementary file 1 [file ijerph-19-13658-s001.zip › ijerph-1889137-supplementary.pdf]

Respiratory tract and eye symptoms in wildland firefighters in two Canadian provinces: impact of discretionary use of an N95 mask during successive rotations.

Nicola Cherry, Natasha Broznitsky, Mike Fedun, Tanis Zadunayski

Supplementary Materials

A) Questionnaires at the start and end of rotations
B) Comments recorded by firefighters allocated N95 masks

Supplementary Material A

Questionnaires at Start and End of Rotation (2021)

The following questionnaires were administered to participants at the start of their rotation shift and at the end of their rotation.

Wildland Firefighter Start of Rotation Questionnaire

We are interested in any symptoms you have now (before stating this rotation). Please put an 'X' on each of the scales below to indicate the most you have been bothered by the symptom in the last 24 hours

Sore itchy or running **eyes** Not at all |———————| Very bothered

Sore, itchy or running **nose** Not at all |———————| Very bothered

Sore or rough-feeling **throat** Not at all |———————| Very bothered

Coughing Not at all |———————| Very bothered

May we now ask you about your smoking history and events that might have exposed you to PAHs?

When did you last smoke a cigarette (including cannabis or vaping) containing tobacco?

☐ In the last 24 hours? If only vaping check here ☐

☐ Not in the last 24hrs, but in the last three months

☐ More than 3 months ago

☐ Never smoked as much as 1 cigarette a day for as much as a year

In the last 24 hours have you:

Spent time with others who were smoking tobacco? ☐ Yes ☐ No

Spent time around a fire pit or BBQ? ☐ Yes ☐ No

Eaten BBQ or smoked meat or fish? ☐ Yes ☐ No

Before today, how many days ago did you last work on a fire? __________________

Is the clothing you are wearing now all clean, or did you wear any of it at a previous fire without it being washed or replaced?

☐ All clean ☐ Some unwashed, specifically the following items:

____

____

Before today, for how many days have you worn this Nomex without it being washed? ________

Date today: _____day______month______year

Wildland Firefighter End of Rotation Questionnaire

A. Symptoms

A1. We are interested in any symptoms you have experienced in the last 24 hours. Please put an 'X' on each of the scales below to indicate the <u>most</u> you have been bothered by the symptom **in the last 24 hours.**

Sore itchy or running **eyes** — Not at all |———————| Very bothered

Sore, itchy or running **nose** — Not at all |———————| Very bothered

Sore or rough-feeling **throat** — Not at all |———————| Very bothered

Coughing — Not at all |———————| Very bothered

A2. Thinking back to the whole of this rotation. Please put an 'X' on each of the scales below to indicate the <u>most</u> you have been bothered by the symptom **at any time during this rotation**.

Sore itchy or running **eyes** — Not at all |———————| Very bothered

Sore, itchy or running **nose** — Not at all |———————| Very bothered

Sore or rough-feeling **throat** — Not at all |———————| Very bothered

Coughing — Not at all |———————| Very bothered

B. Exposure to polycyclic aromatic hydrocarbons (PAHs).

B1. When did you last smoke a cigarette (including cannabis or vaping) containing tobacco?

☐ In the last 24 hours? If only vaping check here ☐

☐ Not in the last 24hrs, but in the last three months

☐ More than 3 months ago

☐ Never smoked as much as 1 cigarette a day for as much as a year

B2. In the past 24 hours have you:

Spent time with others who were smoking tobacco (including cannabis, vaping)? ☐ Yes ☐ No

Spent time around a fire pit or BBQ? ☐ Yes ☐ No

Eaten BBQ or smoked meat or fish? ☐ Yes ☐ No

B3. How many days ago did you last work on a fire? __________________

C. Factors affecting skin exposure/absorption.

C1. Have you worn the same nomex, without washing or replacing it with a clean one, throughout this rotation?

☐ Yes (go to C2) ☐ No

If No, what is the *maximum* number of days during this rotation you wore the nomex without washing or replacing it?___________days

C2. How many days ago (including today) did you put on a clean nomex__________?

C3. During this rotation, how often were you able to shower thoroughly at the end of the work day?

Every day? ☐

Most days? ☐ Please estimate total number of days **missed** ____________

About half the days ☐

Less than half the days ☐

Never or almost never ☐

C4. Were arrangements for showering adequate throughout this rotation? ☐ Yes ☐ No

If no, how could they have been improved? ____

____

C5. During this rotation, how often were you able to put on a complete set of clean clothes after showering (that is clean underwear, socks, pants, shirt/T-shirt)?

Every day? ☐

Most days? ☐ Please estimate total number of days **missed**_________

About half the days ☐

Less than half the days ☐

Never or almost never ☐

C6. Did you sometimes have only a partial change of clothes? ☐ Yes ☐ No

If yes, which items were you most likely to re-wear without washing or replacing?_________

____

C7. For each task below please indicate whether or not you did this at all **during this rotation**

If yes, please put a X to show how often you wore an N95 mask during this task.

	Yes	No			
Initial attack:	☐	☐	Always		Never
Sustained action:	☐	☐	Always		Never
Prescribed fire:	☐	☐	Always		Never
Hazard reduction:	☐	☐	Always		Never
Hot spotting:	☐	☐	Always		Never
Mop up:	☐	☐	Always		Never
Burnout:	☐	☐	Always		Never
Patrol/recon:	☐	☐	Alway		Never
Gridding:	☐	☐	Always		Never
Driving:	☐	☐	Always		Never

Other. ☐ ☐ Always |———| Never
Please Specify:

____

____

____

D. Use of an N95 mask.

If you wore an N95 **at any time during this rotation**, please answer questions D1- D3 below.
If you did NOT wear an N95 mask during this rotation please go to E below, which asks for today's date

D1. How often did you wear a mask for all or part of a day?

Every day? ☐

Most days? ☐ Please estimate the total number of days you did ***NOT*** wear it at all.
_________days

About half the days ☐

Less than half the days ☐

Never or almost never ☐

D2. **If ever worn**, please mark an 'X' on the scales below to tell us about your experience while wearing the mask.

Was it comfortable? Not at all |———| Very much

Did it fit well? Not at all |———| Very much

Did it make breathing difficult? Not at all |———| Very much

Did if make your firefighting work more difficult? Not at all |———| Very much

Did you feel protected from the smoke/ash? Not at all |———| Very much

D3. Do you have any additional comments about wearing the mask?

____

____

____

E. Date today:________day__________month__________year

THANK YOU FOR COMPLETING THIS QUESTIONNAIRE

Supplementary materials B: Comments recorded by firefighters allocated N95 masks

Comments

Communication

- Hard to talk on radio
- Still hard to talk on the radio
- Very hard talking to others
- Difficult to communicate with ground and air assets
- Muffled communication.

Mop-up

- Masks are great for mop-up. A better seal would be good.
- Masks do not fit properly, but are still in my opinion better than not wearing one. Great for mop up, hot-spotting/dry mopping. Better fitting and with an exhale valve would make these masks great
- Masks are great for mop up and hot spotting
- Masks are great for mop up/ hot spotting

Work harder

- Made recovering from heavy breathing difficult. Limited visibility slightly
- Fine for basic activities, difficult when working hard and in higher fire activity
- They suck to wear while on a fire. They hinder my ability to function at 100%
- They suck. Hard to breath thus hard to perform my job
- More in shape (fit) the person is, the easier it is to wear
- Vigorous work makes it very hard to keep on, breathing becomes hard

Other

- perspiration soaks seal and makes mask wet

- Does wearing the mask affect how much CO2 I'm breathing in while working? What affect does this have?

- It seems like they get dirty very quickly. Not sure how long it is actually able to effectively filter air for.

- I used the mask for the worst parts of the season but not much on this (pull)

- Having a beard renders the mask useless. Most often worn in mop up and in heavy smoke.
- Proper N95 fit testing should be done to ensure a proper fit.
- I would rather wear a half face respirator, because I hate disposable masks
